# What can fish brains tell us about visual perception?

**DOI:** 10.3389/fncir.2014.00119

**Published:** 2014-09-29

**Authors:** Orsola Rosa Salva, Valeria Anna Sovrano, Giorgio Vallortigara

**Affiliations:** ^1^Center for Mind/Brain Sciences, University of TrentoRovereto, Trento, Italy; ^2^Dipartimento di Psicologia e Scienze Cognitive, University of TrentoRovereto, Trento, Italy

**Keywords:** perceptual organization, fish, chondrichthyes, osteichthyes, visual system, visual illusions, color constancy, perceptual binding

## Abstract

Fish are a complex taxonomic group, whose diversity and distance from other vertebrates well suits the comparative investigation of brain and behavior: in fish species we observe substantial differences with respect to the telencephalic organization of other vertebrates and an astonishing variety in the development and complexity of pallial structures. We will concentrate on the contribution of research on fish behavioral biology for the understanding of the evolution of the visual system. We shall review evidence concerning perceptual effects that reflect fundamental principles of the visual system functioning, highlighting the similarities and differences between distant fish groups and with other vertebrates. We will focus on perceptual effects reflecting some of the main tasks that the visual system must attain. In particular, we will deal with subjective contours and optical illusions, invariance effects, second order motion and biological motion and, finally, perceptual binding of object properties in a unified higher level representation.

## The fish as a model of object processing in the visual system

Fish represent a highly complex taxonomic group, whose divergence from the other vertebrates is estimated to have occurred approximately 450 million years ago (Kumar and Hedges, 1998). Jawless fish (*Agnatha*) represent one of the oldest vertebrate forms (Foley and Janvier, 1993). Cartilaginous fishes (*Chondrichthyes*), which appeared about 400 million years ago, represent the oldest extant jawed vertebrates and preserve a number of their ancestral traits having evolved at a much slower rate than other classes (Martin et al., 1992). Contrary to mammals and avians, fish do not actually represent a single clade, but a paraphyletic collection of taxa, including jawless, cartilaginous and bony-fish species (Nelson, 2006). Within the bony-fishes, we find the Actinopterygii or ray-finned fishes, that alone represent the largest subclass of vertebrates, comprising of more than 30 thousand species (mostly belonging to the superorder of Teleosts). This great taxonomic diversity within fish species, and the phylogenetic distance that separates fish from other vertebrates, present an invaluable opportunity for the comparative investigation of brain and behavior in an evolutionary perspective. We will here concentrate on the contribution of research on the behavioral biology of fish for the understanding of the evolution of the visual system.

Many fish species rely mainly on vision, using it to guide a wide range of behaviors (Guthrie, 1986; Brown et al., 2011). Not surprisingly, it has been demonstrated that fish have well developed visual capabilities that match those of other vertebrates (von Frisch, 1914; Douglas and Djamgoz, 1990; Vallortigara, 2004; Brown et al., 2011). In the literature we find a number of studies on the perception of shape and color in fish species, showing for example that several Teleost fishes have excellent trichromatic color vision (Beauchamp, 1978), as well as the capacity to discriminate two- and three-dimensional shapes (Schaller, 1926; Herter, 1929, 1930; Hager, 1938; Meesters, 1940; Mackintosh and Sutherland, 1963; Sutherland, 1964; Mark, 1966; Wyzisk, 2005; Wyzisk and Neumeyer, 2007; Siebeck et al., 2009; Schluessel et al., 2012; Gierszewski et al., 2013). Motioperception has also been studied in fish, with a particular attention for model organisms such as zebrafish. Shortly after hatching zebrafish innately respond to movement with a characteristic optomotor response (Clark, 1981; Neuhauss et al., 1999). Different species of fish, from Elasmobranchs to Teleosts, have also revealed sophisticated cognitive abilities in the visual domain, distinguishing various shapes from their mirror image counterparts (Gierszewski et al., 2013) and succeeding in visual categorization tasks (Schluessel et al., 2012, 2014a,b; Schluessel, 2014).

With regards to the physiological substrate of vision, at the peripheral level the functioning of the fish visual system has been extensively studied (especially in morphology and electrophysiology). The great variety of taxonomic groups and ecologic niches that we observe in fish, together with their long evolutionary history, account for the surprising diversity documented in the organization and function of eyes of different species (Douglas and Djamgoz, 1990). In contrast, until recently, less was known about the organization and function of higher visual processing stations in the telencephalon, especially in comparison with other more well-studied taxa. In fish, as in amphibians and sauropods, we do not observe a layered structure resembling the mammalian neocortex, even though of course the general Bauplan of the vertebrate brain is respected (Wullimann, 1997; Northcutt, 2011). In recent years, our knowledge of the brain functioning and neuroecology of various fish groups has greatly increased (Teleosts, Broglio et al., 2011; for Elasmobranchs see Collin, 2012; Yopak, 2012a,b). This has revealed an astonishing variety in the development and complexity of pallial structures in different fish species, sometimes even when considering species belonging to close groups (Mueller et al., 2008; Mueller and Wullimann, 2009; Rodríguez-Moldes, 2009; e.g., Actinopterygii differ from all other vertebrates in that their telencephalon develops by eversion of the lateral walls and has no lateral ventricles; different species however show great variation in the degree of eversion, and thus in the pallial architecture, Nieuwenhuys, 2011). As it has been the case for other non-mammalian vertebrates, in the last decade scientists have started to recognize that fish telencephalon is not composed mostly of basal ganglia (subpallium), but includes wide pallial regions that bear homologies with the mammalian neocortex. These pallial structures potentially serve functions similar to the neocortex, instead of being simply devoted to olfactory processing (Wullimann and Mueller, 2004; Jarvis et al., 2005; Portavella and Vargas, 2005; Rodriguez et al., 2006; Costa et al., 2011). Despite these increasingly recognized homologies, the fish brain has clearly less computational power than what available to the primate cortex (Van Essen et al., 1992; Hansel and Sompolinsky, 1996; Kawai et al., 2001; Hill et al., 2003; Horton and Adams, 2005). Thus, the investigation of the perceptual and cognitive functioning of fish can provide information about the complexity of the neural circuitry required for a given function. This is especially true for those visual phenomena that have been traditionally considered limited to humans and only a few other mammals.

We shall review evidence obtained in different fish species concerning perceptual effects that reflect fundamental principles of the visual system functioning. We will highlight the similarities and differences between distant fish groups and with other vertebrates. Across most animal species the visual system faces similar challenges and must fulfill similar requirements to allow meaningful interaction with physical objects and adaptive responses to the external environment. In subsequent sections of the paper, we will focus on four primary tasks that a functional visual system must attain:

1. Processing of objects as wholes, unified entities, segregated from the background. This is accomplished by visual interpolation processes and grouping mechanisms, whose action is revealed by phenomena such as amodal completion, illusory contours and some optical-geometrical illusions. We shall review evidence of these phenomena in distant fish species, with implications for the evolution of the corresponding neural substrates.
2. Ensuring constant perception of invariant object properties such as size, shape and color, despite the constant modification of the physical (proximal) input reaching the retina, due to changes in viewing distance, perspective and illumination conditions. Fish species have provided an interesting model for the study of the neural implementation of size, color and, recently, shape invariance.
3. Attentional prioritization and effective processing of complex motion information. We shall focus on second order motion and biological motion, two cases of computationally-complex motion processing that have been recently demonstrated in fish species.
4. Binding different object properties, such as shape, color and motion, in a unified higher level representation. In fish as in other vertebrates, in the earlier stages of visual processing these properties are processed by independent channels. Bringing them into a single representation was traditionally considered an extremely challenging task, carried out by areas of the associative cortex, a view that has been challenged by the demonstration of perceptual binding in fish.

## Visual interpolation processes: amodal completion and illusory contours

**Figure 1:**
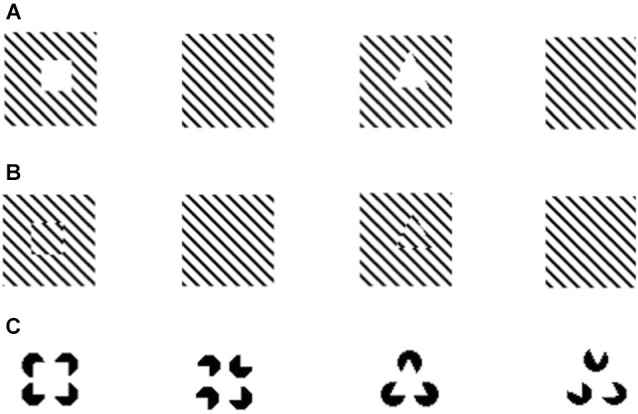
**Examples of illusory contours demonstrated in *Xenotoca eiseni* (Sovrano and Bisazza, 2009)**. After being trained to discriminate different shapes fish were presented with illusory squares and triangles created through interruption of diagonal lines **(A)**; spatial phase shift of diagonal lines **(B)**; or through the Kanizsa displays **(C)**.

Visual illusions are instances of systematic discrepancy between a physical description of distal or proximal stimuli and perception. As such, they provide important insight about how the visual system operates (Bruce et al., 2003). In particular some illusions provide information on how the visual system integrates sensory stimulation into a unified representation (Nieder, 2002). The perception of illusory contours (which are not determined by a contrast gradient in the physical word, Figure 1) and the amodal completion of partially occluded objects are primary examples of the visual system’s ability to interpolate visual information (Kanizsa, 1979). Both of these phenomena reflect grouping mechanisms that promote processing of objects as wholes and underlying neural mechanisms that represent object boundaries regardless of how they are defined in the sensory input (Sekuler and Palmer, 1992; Palmer, 1999; Kellman et al., 2001, 2005; see Nieder, 2002 for a review of neural mechanisms). These traits are likely to have emerged as a consequence of the adaptive need to segregate in a unitary percept partially occluded objects or objects presented through degraded visual information. In fact, form perception is possible because the visual system processes sensory information about shape, color, distance, and movement of objects according to its own system-specific rules (Kandel et al., 2000). Subjective contours are the manifestation of these principles, the action of a network that is predisposed to complete certain figural elements (Kanizsa, 1976; Gerbino and Salmaso, 1987; Purghé and Coren, 1992; Nieder and Wagner, 1999). The application of these processing principles allows the brain to reconstruct contours missing from the retinal image (Nieder and Wagner, 1999) and to selectively merge only some parts of the visual scene (Kandel et al., 2000). When perceiving subjective or amodal contours the visual system’s response is based on assumptions on the likely state of things in the external word, rather than on the actual retinal input (Kanizsa, 1979; Day and Kasperczyk, 1983; Kandel et al., 2000). These assumptions are of course not to be intended as conscious explicit inferences, but rather reflect the action of pre-wired adaptive mechanisms available in the absence of previous experience at the individual level (e.g., Regolin and Vallortigara, 1995).

As we have mentioned above, a similar neural computational mechanism is purported to underlie both modal perception of illusory contours and amodal completion (Kellman and Shipley, 1991; Kellman et al., 2005) (e.g., filling-in mechanisms known in mammals Kellman et al., 1998; Kandel et al., 2000). Comparative research in fish has contributed to support this claim, revealing that species that are sensitive to one of the phenomena tend to also perceive the other (e.g., see Sovrano and Bisazza, 2008, 2009 for redtail splitfin fish).

Moreover, evidence obtained in fish species helped to understand the phylogenesis of this mechanism. The demonstration of susceptibility to amodal completion and illusory contours in this highly diverse taxonomic group, in addition to birds and mammals, suggests a conserved trait that is widespread in vertebrates and inherited from a common ancestor, rather than a case of convergent evolution in the different classes. In this regard it is particularly interesting to consider the high phylogenetic diversity of the fish species that respond to illusory contours and amodal completion. For example, illusory contours are perceived by teleosts as distant as Ostariophysi (redtail splitfin fish, *Xenotoca eiseni*) and Acanthopterygii (goldfish, *Carassius auratus*) (Wyzisk and Neumeyer, 2007; Sovrano and Bisazza, 2009). Surprisingly, while in the study of Sovrano and Bisazza (2009) redtail splitfins were able to recognize also illusory geometric shapes created by phase shifts or by interruption of diagonal lines, the goldfish tested by Wyzisk and Neumeyer (2007) could not recognize phase-shifted illusory shapes. However, this discrepancy may be due to a methodological problem in the stimuli of Wyzisk and Neumeyer, which consisted of very thin lines, reducing the strength of the illusory perception.

**Figure 2:**
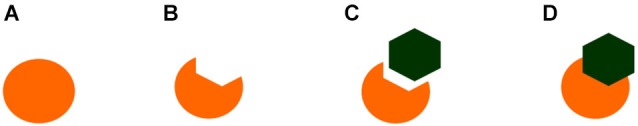
**Examples of the stimuli employed by Sovrano and Bisazza (2008) to demonstrate amodal completion of partially occluded objects in *Xenotoca eiseni***. In figure **(A)** and **(D)**, but not in **(B)** or **(C)**, the circle is perceived as being complete or amodaly completed behind the occluder.

Similarly, amodal completion is observed in two species of Acanthopterygii (*Variola louti* and *Scarus niger*), in addition to the redtail splitfin fish (Sovrano and Bisazza, 2008; Darmaillacq et al., 2011; Figure 2). Recently it has been found that even cartilaginous fish (bamboo sharks, *Chiloscyllium griseum*) are susceptible to amodal completion and illusory contours (Fuss et al., 2014), despite being the oldest extant vertebrates and having conserved many of their ancestral traits (Martin et al., 1992).

Remarkable similarities in the distinctive traits of the visual interpolation effects observed in humans and in fish species further support the presence of a conserved mechanism. For example, both in goldfish and in human beings the perception of Kanizsa figures is disrupted by the superimposition of black lines (von der Heydt, 2004; Wyzisk and Neumeyer, 2007). This result, in humans, is considered consistent with the idea that neurons at the level of V2 are responsible for the perception of illusory contours. In primates, 60% of V2 neurons respond to illusory contours (von der Heydt et al., 1984), the same percentage observed in the visual Wulst of owls (Nieder and Wagner, 1999)^1^. This seems to suggest that forebrain structures should provide the neural basis of these phenomena in fish as well. However, a recent study in pigeons challenged the view that forebrain structures are mainly responsible for the perception of illusory contours. This study showed that pre-tectal neurons are capable or responding to real and subjective contours alike (Niu et al., 2006). Whether similar mesencephalic mechanisms are involved in the perception of illusory contours in fish is a question that calls for empirical investigation, in order to shed light on the phylogenesis of this trait in different classes.

## Geometrical illusions and hierarchical processing

Another widely studied class of perceptual phenomena, associated with grouping mechanisms, is that of geometrical size illusions, in which properties of a target stimulus, such as length, width, or diameter, are distorted by the surrounding context, providing an important tool for the study of perceptual integration of local elements into global context. Both mammalian and avian species are susceptible to geometrical illusions. For example, let us consider the Ponzo perspective illusion, in which two identical horizontal segments look different in length in the context of two converging lines, with the segment that is closer to the point of convergence appearing longer than the other. This illusion has been demonstrated in horses (Timney and Keil, 1996), monkeys (Bayne and Davis, 1983; Barbet and Fagot, 2002; see also Fujita, 1996), chimpanzees (Fujita, 1997), and pigeons (Fujita et al., 1991, 1993; Fujita, 2006; Nakamura et al., 2006, 2009). Similarly, the Müller-Lyer illusion (in which a line segment with two arrows facing outwards at the end appears longer than one with arrows facing inwards) deceives capuchins and rhesus monkeys (Suganuma et al., 2007; Tudusciuc and Nieder, 2010), as well as gray parrots (Pepperberg et al., 2008) and ring doves (Warden and Baar, 1929).

**Figure 3:**
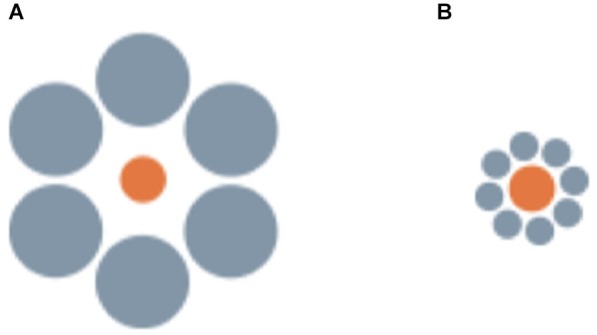
**Examples of the stimuli employed by Sovrano et al. (submitted; Sovrano, 2014) in *Xenotoca eiseni***, **to investigate the Ebbinghaus illusion, in which a central circle surrounded by large circular inducers is perceived as smaller than an identical circle surrounded by small inducers**.

A less clear case seems to be that of the Ebbinghaus illusion, in which a central circle surrounded by large circular inducers is perceived as smaller than an identical circle surrounded by small inducers (Figure 3). This is one of the strongest geometrical illusions in humans (Ebbinghaus, 1902), but seems absent or even reversed in non-human primates (Parron and Fagot, 2007) and birds (pigeons and bantams Nakamura et al., 2008, 2014). In humans this illusion reflects the action of grouping mechanisms (as revealed by the fact that the strength of the illusion is influenced by the distance between the central target and the surrounding inducers, Roberts et al., 2005). Thus, the difficulty of obtaining evidence of its presence in non-human species seems to indicate a radical difference in the functioning of these mechanisms between our species and non-human animals. It has been also suggested that the neural circuitry underlying to the perception of the Ebbinghaus illusion might have evolved recently in mammals or even in the primate lineage (Parron and Fagot, 2007; Nakamura et al., 2008). However, this would be surprising given the evidence of widespread susceptibility to amodal completion and illusory contours (reflecting the action of interpolation and grouping mechanisms) in vertebrates ranging from mammals to different fish classes (see Section Visual interpolation processes: amodal completion and illusory contours). Notably, the three studies that failed to demonstrate human-like perception of the Ebbinghaus illusion in non-human animals all involved training and testing of the animals with touch screens, which require the subjects to perform a manipulative response (touching or pecking) and, in the case of pecking, also force a very close view of the stimuli when emitting the response. In humans, the Ebbinghaus illusion is also reduced when tested through motor tasks requiring a manipulative response (Aglioti et al., 1995; Danckert et al., 2002). This is in line with an involvement of the human neocortex, where the two independent neural pathways, the dorsal and the ventral stream, are responsible for visual awareness and for action control (Goodale and Milner, 1992). Moreover, forcing the subjects to inspect the stimuli from a close distance could have prompted them to pay attention only to the central target or to its immediate proximity. This could have caused the direction of the illusion to be reversed, transforming it into an assimilation illusion (analogous to what is observed in humans when the distal portion of the inducers is not visible, Oyama, 1960; Weintraub, 1979). In support of this interpretation, human-like perception of the Ebbinghaus illusion was reported in a recent study with domestic chicks that employed a more naturalistic training procedure, based on incidental learning, and a test procedure allowing the animals to observe the stimuli at a freely chosen looking distance (Rosa Salva et al., 2013). In this study, subjects that were habituated to finding food behind a screen depicting, for instance, a small orange circle, and then tested with the illusory configurations, preferred to look behind the screen depicting the perceptually smaller circle. Thus, when appropriate procedures are used, avian species are also found to be susceptible to the Ebbinghaus illusion. Most interestingly, using similar naturalistic training and testing procedures, we have been recently able to demonstrate the perception of this geometric illusion in teleost fish, finding that redtail splitfin fish also perceive the Ebbinghaus illusion as a contrast illusion (Sovrano, 2014; Sovrano et al., submitted). Different groups of fish were trained to locate the exit marked by a bigger or a smaller orange circle, in order to escape from the test arena and rejoin conspecifics. When tested with the illusory configurations, fish trained on the bigger orange circle preferred to approach the circle that appeared perceptually bigger in the Ebbinghaus display (i.e., the orange circle surrounded by small gray inducers). Similarly, fish reinforced on the smaller orange circle preferred to approach the illusory display in which the central circle appeared perceptually smaller (being surrounded by big inducers).

**Figure 4:**
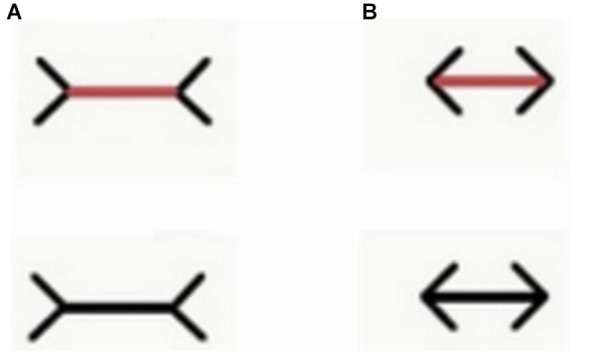
**Examples of the stimuli employed by Sovrano et al. (in preparation; Sovrano, 2014) in *Xenotoca eiseni***, **to investigate the Müller-Lyer illusion, in which a line with two arrow-shaped inducers at its ends facing outwards (A) appears longer than an identical one with inducers at its ends facing inwards (B)**. In a control condition (upper part of this figure) the line is of a different color than the inducers (the line is red while the inducers are still black). In this condition the line was red also during training.

Moreover, in contrast with previous unsuccessful attempts with goldfish (Wyzisk, 2005; but see Herter, 1930 for an earlier report with small sample size, finding discrepant results), in a recent study it has been demonstrated that teleost fish (redtail splitfin) can perceive the Müller-Lyer illusion (Müller-Lyer, 1889), like humans and other vertebrates do (Sovrano, 2014; Sovrano et al., in preparation; Figure 4). Fish were trained to discriminate between two lines of different length. Reinforcement was provided by the possibility to rejoin conspecifics, escaping from the test arena through an exit, recognizable since it was marked by a longer or a shorter line. Then fish were presented with two lines of the same length with two arrow-shaped inducers facing inwards or outwards. Subjects chose the stimulus that, on the basis of the perception of the Müller-Lyer illusion, appeared deceptively larger or smaller, consistent with the condition of training. Curiously enough, another existing study investigating the perception of the Müller-Lyer display in a fish species revealed that bamboo sharks are not deceived by this illusion (Fuss et al., 2014). Elasmobranchs (sharks and rays) belong to the class of cartilaginous fishes. Thus, a possibility for reconciling these contradictory results would be to hypothesize that cartilaginous and bony fish differ in their ability to perceive geometric illusions in general, or the Müller-Lyer display in particular. This would have important implications for our understanding of the phylogenesis of the visual system, indicating that the neural substrate for the perception of this geometrical illusion could have evolved after the separation of cartilaginous and bony fish. Due to the great phylogenetic distance between sharks and teleost, and in particular to divergent developmental processing (Northcutt, 1977; Wullimann and Mueller, 2004; Nieuwenhuys, 2009), major differences can be observed in the brain organization between these different classes, justifying the idea of a real dissociation of perceptual mechanisms available to cartilaginous and bony fishes. Notably, bamboo sharks tested in the same study were able to perceive Kanizsa figures and illusory contours (Fuss et al., 2014). This could indicate that the perception of subjective contours depends on conserved neural mechanisms that emerged earlier in phylogenesis than those underlying to the perception of the Müller-Lyer illusion, which could have been evolved after the divergence of cartilaginous and bony fish. Another possible interpretation would be, of course, that the mechanism allowing perception of subjective contours has an adaptive value in a wider range of species, including Elasmobranchs, and has thus been evolved independently multiple times. However, caution is needed before venturing too far with evolutionary interpretations on the basis of data collected only in two species and in two studies that employed different training methodologies. In the study of Fuss et al. (2014) sharks were food reinforced for pressing their nose against the wall just below/onto the positive stimulus, implying a very close inspection of the stimuli. On the contrary, red tail splitfins learned to use line length to orient in the test tank and locate its exit. Also, for the bamboo sharks tested by Fuss and colleagues, learning the line-length discrimination task resulted much more difficult than the other tasks trained in this study (e.g., in Experiment 3a only three sharks out of eight managed to learn to discriminate three pairs of lines based on their length, and none of them was able to learn the fourth pair proposed). Bamboo sharks seem thus to be not very sensitive to differences in line lengths in general, even when these differences are real rather than illusory. Interestingly, the goldfish trained by Wyzisk (2005), who also did not seem to perceive the Müller-Lyer illusion, had an even worse performance in learning the line discrimination task than the bamboo sharks. It is thus possible to hypothesize that the illusion itself could affect also sharks and goldfish, but that its extent, in the version tested by Fuss et al. (2014) would not be enough to create a sufficiently pronounced difference in perceived line length to reliably sustain performance. In fact, one of the six individuals tested in Experiment 3b seemed to be affected by the illusion, systematically choosing the display with inverted arrowheads. Also in the human species the Müller-Lyer illusion evokes only a slight deception and does not affect all individuals (Rivers, 1901; Segall et al., 1966; Berry, 1968), revealing again a striking similarity between the mechanisms present in very distant species.

**Figure 5:**
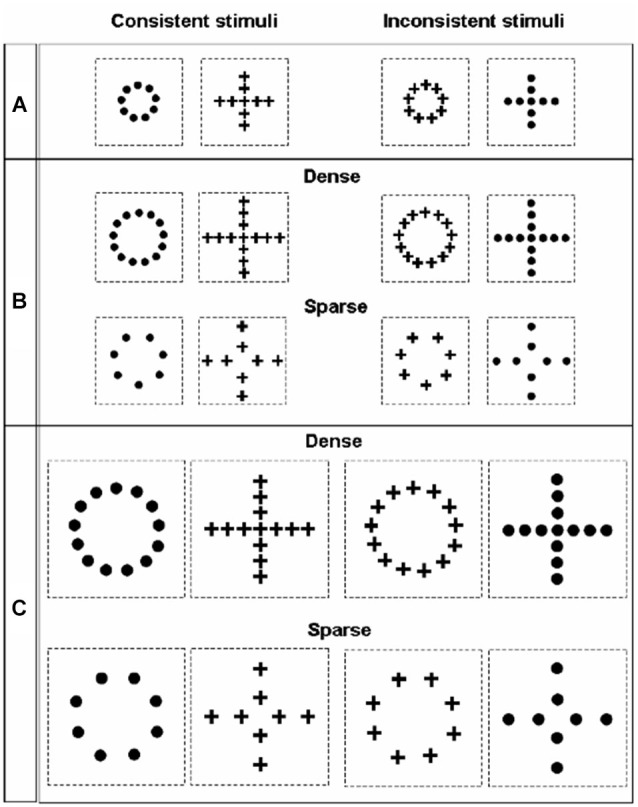
**Stimuli employed by Truppa et al. (2010) to investigate processing of hierarchical stimuli in *Xenotoca eiseni***. On the left side are the consistent stimuli presented, in which the same shape is represented at the global and local level; on the right side are the inconsistent stimuli, in which the shape information provided by the local and the global level conflict. Across the three different conditions (**A**, **B** and **C**), stimuli differed in absolute size and in the density of the local elements.

The perception of geometrical illusions, such as those created by the Ebbinghaus or Müller-Lyer displays, has been often linked to the tendency of a species or of an individual to apply either a more global or a local processing strategy (Parron and Fagot, 2007; Nakamura et al., 2009, 2014; Rosa Salva et al., 2013). In fact, the tendency of the visual system to process visual configurations as wholes, rather than focusing on single details in isolation, allows contextual elements surrounding the target object to distort its perception. Since the seminal work of Navon (1977, 1981), hierarchical stimuli have been used to investigate the interplay of local and global processing in different species and in different tasks. In hierarchical stimuli a bigger global configuration is created by the juxtaposition of many smaller figures (Figure 5). The human species seems to be endowed with a remarkably globally-oriented perceptual style that makes us see “*the forest before the trees*” (Navon, 1977). That is to say, in most situations we tend to prioritize the processing of the bigger configuration (global level), rather than of the smaller figures composing it. On the contrary, evidence obtained in non-human primates and in some other species seemed to indicate a general tendency to prioritize the local information about the individual shapes, bringing some authors to suggest that a globally-oriented perceptual style would be limited to humans, with the possible exception of some great apes (e.g., Fagot and Deruelle, 1997; Deruelle and Fagot, 1998; Cavoto and Cook, 2001). Over the years evidence accumulated indicating that this is likely to be an extreme oversimplification. For instance, depending on the context of the current task and on viewing conditions, humans can display a locally oriented perceptual style (Kimchi, 1992), whereas pigeons (traditionally considered an exemplar case of locally-oriented perception, Cerella, 1980; Cavoto and Cook, 2001) are able to flexibly switch the focus of their attention between the local and the global level (Fremouw et al., 1998, 2002). Notably, the first clear demonstration of global dominance in the perception of hierarchical stimuli in non-human animals has been obtained few years ago in red tail splitfin fish trained according to the same general procedure described above for the demonstration of the Ebbinghaus and Müller-lyer illusions (Truppa et al., 2010). Again, this suggests that, when ecologically valid training and testing procedures are used, it is possible to demonstrate remarkable similarities in the grouping mechanism employed by the visual system of fish and of other vertebrates, despite great phylogenetic distance.

## Invariance effects: is cortex needed for invariant color perception?

Some of the visual illusions mentioned above have been hypothesized to reflect the action of adaptations evolved to ensure invariance in perception, despite huge variations in the physical parameters of the retinal input (e.g., Gregory, 1963; but see Humphrey and Morgan, 1965). For example, the Ponzo perspective illusion might involve the same mechanisms that give rise to the perception of size invariance (the tendency to perceive the absolute size of a known object, despite differences in the size of the pattern projected on the retina when the object is viewed from various distances) (Gregory, 1963; Fujita, 1996; but see Georgeson and Blakemore, 1973; Newman and Newman, 1974). Research in fish species has a long tradition for the investigation of size invariance, that has been demonstrated repeatedly in Actinopterygii species (Herter, 1930; Douglas et al., 1988; Schuster et al., 2004; Frech et al., 2012). In addition to that, more recently, form invariance has also been shown in Malawi cichlids (*Pseudotropheus* sp., Schluessel et al., 2014b; see Wood, 2013 for evidence that the ability to form viewpoint-invariant representations of 3D objects represents a core and experience-independent cognitive trait).

Here we will concentrate on an exemplar case, describing the contribution of fish as an animal model of the physiological basis of color invariance, the mechanisms by which the visual system recognizes an object as having a consistent color regardless of the spectral composition of the light reflecting from it at a given moment (see Foster, 2011 for a comprehensive review on this phenomenon). Simultaneous color contrast is a related phenomenon to color invariance. In this case the perceived hue of a small visual region is altered by the presence of a colored surround: gray regions are perceived as of a hue complementary to that of the surround, whereas colored regions assume a hue “away” from that of the surround (Graham and Brown, 1965).

At the behavioral level, research on a very popular model organism, the goldfish, has demonstrated that this species is able to make color-constant judgments, implying the perception of color invariance (Ingle, 1985; Neumeyer et al., 2002). Simultaneous color contrast has been demonstrated in various Teleost species, including goldfish and other two Cyprinids (*Tinca vulgaris* and* Barbus paripentazona*), two Cichlid (*Hemichromis bimaculatus* and* Pterophyllum scalare*), the three-spined stickleback and a Gasterosteidae (*Gasterosteus aculeatus*) (Herter, 1950; Dörr and Neumeyer, 1997).

One of the most relevant models for understanding how the visual system could implement color invariance and color contrast effects is the *retinex* model by Edwin Land (Land, 1959a,b, 1983; McCann and Benton, 1969; Land and McCann, 1971; Land et al., 1983). This model theorizes a mechanism that computes, for each visual region, the relations between spectral features, based on the comparison of the lightness information provided by each photoreceptor system, and then collates them between distant regions^2^. The term *retinex* was coined combining the words retina and cortex, due to the uncertainty on the location of the neural substrate for these computations. Neural mechanisms underlying to color invariance have been identified over the years: partial chromatic adaptation (within-class cone adaptation), spatial comparisons of cone and cone-opponent signals and invariant responses. These operate at different levels in the visual system. An incomplete chromatic adaptation takes place in the retina’s horizontal cells and in the geniculate nucleus (Creutzfeldt et al., 1991a,b; Lee et al., 1999). In line with what hypothesized by Land, recordings in the retina of goldfish revealed that the horizontal-cell network modulates the processing of cone signals so as to render the ratio of the responses of the three cone-systems stable across illumination conditions (Kamermans et al., 1998; Kraaij et al., 1998).

However, retinal adaptation mechanisms act locally and are not sufficient to fully explain the phenomena associated with color invariance. It is thus believed that, in the primate visual system, computations over spatially extended regions accounting for non-local effects take place in the primary visual cortex V1 or at higher stages of processing (e.g., V4) (Foster, 2011). Two different mechanisms for color-invariance have been discovered in primate V1. The first one is still involved in computations over less spatially extended regions and is based on double-opponent neurons that present both color and spatial opponency. This allows the computation of local ratios of cone activity, in line with what predicted by the *retinex* model. Double opponent cells, before being identified in the primary visual cortex of macaques (Conway, 2001; Conway and Livingstone, 2006), were first discovered in the goldfish retina (Daw, 1967), providing a neural substrate that could partially support color invariance in this species. However, this mechanism can compute the relations between reflectance of nearby areas only. It is thus not sufficient to fully explain color invariance, which involves effects over more spatially extended regions (Land, 1983; Land et al., 1983). In monkeys, networks supporting such comparisons have been identified in V1 and V4 (see Foster, 2011 for a review). In fish there are no known cortex homologs, prompting a question about which neural substrate supports this shared phenomenon within such a differently organized visual system.

## Second order motion and biological motion

Up to now we have explored the perception of static visual objects, with particular attention to grouping mechanisms ensuring the perception of objects as units segregated from the background and to mechanisms that allow to perceive objects’ properties as constant, despite the continuos variation of the physical input reaching the retina. We will now examine the contribution of research on fish species to our understanding of the mechanisms underlying the perception of two peculiar kinds of motion, second order motion and biological motion. We want to warn the reader, however, that this is somewhat an arbitrary distinction that we follow for the sake of argumentation. For instance, it is well known that motion is an extremely important cue for object-background segregation (biological or agentive motion represents a paradigmatic case on this regard Bertenthal and Pinto, 1994; Oram and Perrett, 1996; Giese and Poggio, 2003; Ibbotson, 2007; Nishida, 2011).

Objects that are moving in space are changing their current state and need to be more closely monitored than static objects. Immediate recognition and effective processing of movement in a visual scene is thus crucial for survival and widespread in animal species. On the contrary, only vertebrates having a more sophisticated visual system (i.e., an elaborated cortex, such as that of mammals), were traditionally supposed to be able to perceive second order motion (Ohzawa, 1999). Second order motion is a peculiar type of motion impression elicited by stimuli in which only second-order features, such as contrast, texture or flicker, are moving (also known as non-Fourier motion) (Ramachandran et al., 1973; Chubb and Sperling, 1988; Cavanagh and Mather, 1989). There is electrophysiological, psychophysical and neuropsychological evidence that, in the cortex of mammals, second-order motion is carried out by a dedicate stream (Albright, 1992; Zhou and Baker, 1993; Smith et al., 1998; Baker, 1999). This supported the view according to which the perception of second-order motion would represent an instance of “higher level” motion processing, limited to primates and few other mammals. Despite that, we now know that zebrafish larvae show an optomotor response to motion stimuli that is qualitatively similar to what is observed in primates, reacting in the same way to first- and second-order motion (Orger et al., 2000; see Theobald et al., 2008 for subsequent evidence of second-order motion perception in invertebrates). This strongly undermines the idea that a primate-like organized visual cortex is necessary to perceive second-order motion, suggesting that this is already processed in earlier stages of vertebrates’ visual system (possibly even on the basis of retinal sensitivity to some second-order features, Shapley and Victor, 1978; Demb et al., 2001). However, it is also possible to hypothesize that similar computations to those occurring in the primate cortex to support the perception of second-order motion are carried out by circuitry located in pallial structures of the fish telencephalon (see Jarvis et al., 2005 for a review on the homologies between non-mammalian pallium and mammalian neocortex).

Not all forms of motion are equally relevant for survival: objects belonging to biologically relevant categories, such as conspecifics, preys and predators, can be recognized thanks to the presence of specific movement patterns, typical of animate creatures in general or of a given species in particular. Humans’ extreme sensitivity to the motion of biological creatures (biological motion) has been revealed using the so called point-light displays (PLD; Johansson, 1973). In these stimuli only a dozen of isolated light-points are visible, strategically placed on the major limb joints of a moving person (or animal), presented on an otherwise homogeneous background. As a consequence, PLD provide very little information about the shape or outline of the moving figure, presenting selectively the motion information. Despite the very sparse visual information available in PLD, as soon as these are put in motion, the impression of a moving animate creature is immediately and inevitably elicited in human adults. Human observers are also able to extract rapidly and effortlessly a large amount of information from PLD of biological motion, even in conditions of degraded visual presentation (Runeson and Frykholm, 1983; Bertenthal and Pinto, 1994; Neri et al., 1998; Sumi, 2000; Troje, 2002; Thurman and Grossman, 2008; Alaerts et al., 2011; Sokolov et al., 2011; Pavlova, 2012; Krüger et al., 2013). Specialized neural circuits for the processing of biological motion have been found in the temporal cortex of human and nonhuman primates (in the superior temporal sulcus, STS, Oram and Perrett, 1994; Grossman et al., 2000; Vaina et al., 2001; Jastorff et al., 2012). This cortical specialization emerges during ontogenesis through the interaction of predisposed mechanisms that prioritize the processing of some specific motion features typical of animate creatures and of the extensive expertise we gain by constant exposure to and processing of this sort of stimulus. In fact, the ability to recognize biological motion depicted in PLD, and the tendency to pay preferential attention to this stimulus, is already present in newborn infants (Simion et al., 2008). Most interestingly, analogous abilities and predispositions to process semi-rigid motion had been previously reported in visually naive newly hatched chicks and quails (Yamaguchi and Fujita, 1999; Regolin et al., 2000; Vallortigara et al., 2005; Vallortigara and Regolin, 2006), suggesting the presence of conserved mechanisms in distant vertebrate species (Johnson, 2006; Troje and Westhoff, 2006; Vallortigara, 2012). Conditioning procedures have been used to prove that also other species of mammals and avians can be trained to discriminate biological motion, which could support the idea of homologous mechanisms (Perrett et al., 1990; Omori and Watanabe, 1996; Dittrich et al., 1998; Tomonaga, 2001; Troje and Aust, 2013). However, one of the most remarkable features of human perception of biological motion is the fact that processing of PLD occurs in an effortless and preattentive manner (e.g., Thornton and Vuong, 2004). To understand whether similar mechanisms are employed also by non-human species it is important to test the presence of spontaneous responses to biological motion stimuli. Until recently, galliformes were the only species in which researchers demonstrated a spontaneous response to biological motion resembling what is observed in humans, with the possible exception of female marmosets (Brown et al., 2010). Nothing at all was known about the ability to perceive biological motion in classes other than mammals and avians. To fill this gap, Nakayasu and Watanabe (2014) exploited the spontaneous tendency of medaka fish (*Oryzias latipes*, another member of the class of Actinopterygii, family Adrianichthyidae) to increase shoaling behavior when seeing moving conspecifics. This indicates that visual mechanisms for the detection of biological motion could be evolutionarily more conserved than previously thought. In this study, medaka fish spent significantly more time swimming along a screen on which they could see a PLD of a swimming conspecific than along a screen on which a PLD of a rigid motion was visible. In addition, medakas proved to be able to discriminate different kinds of biological motion, preferring the motion pattern of conspecifics to human motion and being particularly sensitive to the smoothness and the speed of the movement. This is particularly relevant since, also in our species, the speed of movement can drastically alter the perception of biological motion, with abnormal speeds giving the impression of unnatural (e.g., robotic or moon-walk) movements (Barclay et al., 1978). Moreover, both humans (Kozlowski and Cutting, 1977; Barclay et al., 1978; Cai et al., 2011) and the fish tested by Nakayasu and Watanabe (2014) seem to be more affected if the movement sequences were slowed down than if velocity was increased.

## Binding of multiple properties of visual objects in a unified representation

In the first part of this review we have concentrated mainly on early visual processes that, starting from a fragmented retinal input, support the creation of a unitary object-percept with invariant properties (e.g., perceptual grouping mechanisms that ensure the processing of an object as a whole, involved in the perception of subjective contours and geometric illusions and possibly in invariance-effects Sekuler and Palmer, 1992; Palmer, 1999; Kellman et al., 2001, 2005). However, in order to interact effectively with objects in the real word, organisms must conduct also more advanced sensory processing that allows them to bind the multiple properties of a given object into a unified higher-level representation. So, after an initial stage of processing carried out by specialized detectors responding selectively to different properties, such as shape, color and movement (Zeki and Shipp, 1988), the visual system must perform the challenging task of perceptual binding in order to allow adaptive behavior in the real world (Treisman, 1996; Roskies, 1999; Wolfe and Cave, 1999). Computationally, binding is considered a highly demanding task, requiring sophisticated neural circuitry to subtend it. Together with the fact that conjunction tasks seem to be particularly difficult for non-human primates (Smith et al., 2004), the absence of clear-cut evidence of this ability in invertebrates and in vertebrates with “simpler” nervous systems, supported the view that only the mammalian cortex (Zeki and Shipp, 1988; Shafritz et al., 2002; Robertson, 2003; Botly and De Rosa, 2009; DiCarlo et al., 2012) or the avian pallium (Cook, 1992; Blough and Blough, 1997; Jarvis et al., 2005; Katz et al., 2010) could provide a neural substrate with enough computational power for binding (Shettleworth, 2008). In the monkey brain, for example, a higher-level associative region (the superior temporal polysensory area, STPa) contains neurons whose response is driven by a conjunction of the properties of form and motion of walking agents (Van Essen et al., 1992; Oram and Perrett, 1996). Given the seemingly universal adaptive value of the capacity to bind multiple object features in a unified representation, however, it would be surprising that no other complexing-behaving animals, outside the mammal and avian classes, evolved this capability. In fact, earlier reports of binding-like abilities in invertebrates and anuran species (Ewert et al., 1979; Schubert et al., 2002) were recently followed by the demonstration that zebrafish can use feature-binding to direct their shoaling behavior (Neri, 2012; in order to demonstrate true perceptual binding, the animal must for example discriminate between two multiple-objects sets, each set containing both features in different objects, with the sole cue for discrimination being the way in which the two features are combined in the same visual object, Shepard et al., 1961; Treisman, 1996; Wolfe and Cave, 1999). In this study zebrafish spontaneously chose to associate with a “natural” movie of swimming conspecifics rather than with a backward version of the movie, while they did not react to another violation that also created an unfamiliar visual scene (movie presented upside down). In the backward movie, movement and shape information were both still present and virtually unaltered, but were inconsistent with each other. To recognize the original movie from the backward one fish needed to integrate form and motion, performing a conjunction task on two attributes that, in primates, are processed by different cortical regions (Zeki and Shipp, 1988; see Sajovic and Levinthal, 1982; Klar and Hoffmann, 2002; Masseck and Hoffmann, 2008, 2009; for evidence of dedicated centers for processing motion information in fish species). This result was then replicated in the same study (Neri, 2012) with computer generated stimuli that were more controlled, even though less natural: an image representing a side view of a zebrafish was moved along a linear trajectory, which could be either consistent or inconsistent with the orientation of the image of the zebrafish (the direction toward which it was facing). As long as a sufficient number of individuals was depicted in this artificial animation, zebrafish were able to direct their response on the basis of the conjunction of motion direction and shape orientation, even when stimuli were constructed using images of another species (needlefish, *Xenentodon*) or when only the frontal part of a zebrafish image was visible.

The implications of these results for our understanding of the way the visual system supports such sophisticated operations are apparent if we consider the vast disparity in available circuitry between primate and teleost (Van Essen et al., 1992; Hansel and Sompolinsky, 1996; Kawai et al., 2001; Hill et al., 2003; Horton and Adams, 2005). This means that the computations necessary for supporting perceptual binding need much less complex neural circuitry than we previously believed (Treisman, 1996; Shafritz et al., 2002; Robertson, 2003). Interestingly, a recent work on imprinting in domestic chicks revealed that these newborn and visually naive subjects spontaneously bind color and shape features into integrated representations at the onset of their experience with visual objects (Wood, 2014), suggesting the presence of a core mechanism devoted to this fundamental task.

## Conclusive remarks

We have reviewed studies that reveal the mechanisms used by the visual system of fish for adaptive object perception. The fundamental functioning principles that allow the appreciation of objects as unified entities, segregated from the background and characterized by invariant properties seem to be shared between species belonging to distant vertebrate classes, including the oldest extant jawed vertebrates. Moreover, Actinopterygii belonging to two different orders are able to perceive second-order motion and biological motion, whose perception in humans is ascribed to the action of specialized cortical areas, and to bind motion and shape properties of a single object in a higher order representation. Perceptual binding, in particular, is intimately linked to higher-level cognitive phenomena such as attention (Treisman, 1996; Robertson, 2003) and has been traditionally considered a computationally challenging task, requiring the full power of the mammalian neocortex.

One of the most important implications of these results is that they challenge the assumption that only the mammalian neocortex (or the avian pallium, Jarvis et al., 2005) has the computational power required to perform the sophisticated operations needed to perceive some of the above mentioned phenomena. The evidence reviewed in this paper must be interpreted in the context of the increasingly recognized presence of pallial structures in the fish telencephalon (e.g., Mueller and Wullimann, 2005, 2009). Nevertheless, the undeniable disparity in available circuitry between primates and Teleosts still needs to be considered (Van Essen et al., 1992; Hansel and Sompolinsky, 1996; Kawai et al., 2001; Hill et al., 2003; Horton and Adams, 2005). Existent studies in fish have already given insight in the neural mechanisms that support some of these shared abilities (e.g., in the case of color invariance), providing a most fruitful ground for further investigation. Another crucial aspect highlighted by research in fish is the similarity in the characteristics of the effects observed in distant classes of vertebrates. For example, both in fish and in humans the perception of Kanizsa figures is disrupted by the same manipulation (von der Heydt, 2004; Wyzisk and Neumeyer, 2007), and the perception of biological motion is similarly affected by changes in speed (Kozlowski and Cutting, 1977; Barclay et al., 1978; Cai et al., 2011; Nakayasu and Watanabe, 2014). These remarkable similarities may indicate an analogous organization, in distant vertebrates, of the neural circuitry involved in these perceptual effects.

On the basis of the above mentioned evidence that suggest “cortical-like” computational circuitry in fish, we can identify some important venues for future research. First of all, it is necessary to increase our knowledge of the organization and origins of the pallial structures in the fish telencephalon. Only by describing in greater detail the homologies between these structures and those composing the mammalian neocortex, we will be able to fully grasp the implications of the behavioral similarities that we have described here. A very promising approach on this regard is that offered by Mueller and Wullimann (2009), who used the zebrafish as a genetic model to search for developmental similarities between Teleosts and mammals, with a focus on early gene expression. These authors propose that the telencephalon of teleosts has evolved by partial eversion, recognizing homologies with all four mammalian pallial areas. In the light of the principle that recognition of homologies is independent of function and connectivity, we face some intriguing related questions. For example, are these similar perceptual functions implemented by homologous structures? Do these similar functions require structurally similar circuits sharing some specific patterns of connectivity? And, going back to behavioral research, what is possible to do with such brains? What is the role of homologies and structural analogies in the determination of the cognitive functions available to an organism?

Fish are an excellent model to investigate perceptual phenomena, not only for their great taxonomic diversity and peculiarly organized telencephalon, but also for the presence of sophisticated visually guided behavior, allowing one to investigate not only perceptual organization, but also higher cognitive visual functions (Schluessel et al., 2012, 2014a,b; Gierszewski et al., 2013; Schluessel, 2014). In addition to being amenable to traditional training procedures, fish perceptual abilities can be investigated also through more naturalistic incidental learning tasks allowing the animal to freely choose the viewing distance from the stimuli (Truppa et al., 2010; Sovrano et al., submitted; in preparation). On this regard it is important to consider the evidence that we have summarized on the perception of the Ebbinghaus illusion, of the Müller-Lyer illusion and on the processing of hierarchical stimuli (see Section Geometrical illusions and hierarchical processing). These three cases beautifully exemplify the importance of the availability of a number of procedures that can be employed in the same species. This possibility is a necessary prerequisite for a meaningful comparison of the results obtained in different species. We have, in fact, seen that the task-context may actually account for the apparent inter-species differences observed in the susceptibility to perceptual phenomena. In addition to advocating caution with the interpretation of evidence obtained in very diverse settings, we can also propose a venue for further research. Future studies should systematically explore, on the same set of animal models, the effect of the different tasks that are typically applied to different species. For example, it would be interesting to adapt to fish species the touch screen/skinner box procedures that are usually employed with pigeons and other birds. Fish can be trained to respond by touching the stimuli or pressing a button in order to obtain a food reward in the close proximity of the visual display. In this case, would they flexibly change their response similarly to what is seen in avian species? Would they adopt a more locally oriented perceptual style and a smaller attentional focus? It is interesting to note that the bamboo sharks tested by Fuss et al. (2014), which did not seem to perceive the Müller-Lyer illusion, were trained to respond by pressing their snout on the stimuli. Unfortunately, this is the only study that investigated the perception of this illusion in a cartilaginous fish species. We are thus unable to draw firm conclusions from this evidence, pointing once again to the need of a systematic investigation of this issue.

Most interestingly, recent studies have also started to exploit fish spontaneous shoaling behavior (Neri, 2012). This offers a great opportunity to study homologies in phenomena such as biological motion, whose perception in humans stands out for occurring in an effortless and preattentive manner (e.g., Thornton and Vuong, 2004). Indeed, spontaneous social responses to biological motion have been shown in naive chicks (Vallortigara et al., 2005; Vallortigara and Regolin, 2006), and, recently, also in medaka fish (Nakayasu and Watanabe, 2014). This highlights another promising venue for future research, which could put the study of perceptual processes and of their neural bases in the context of social behavior. A similar approach has been used with galliform chicks. Research in domestic chicks revealed that they are endowed with a set of unlearned perceptual and cognitive mechanisms that predispose them to appropriate social interactions. These early mechanisms are, thus, tightly linked to the evolutionary pressures posed by the social environment. Overall, chicks’ perceptual and cognitive predispositions ensure preferential processing of stimuli associated with conspecifics, direct imprinting toward appropriate stimuli, maintain the brood cohesion and facilitate social learning (e.g., Regolin and Vallortigara, 1995; Johnston et al., 1998; Rosa Salva et al., 2009, 2010, 2011, 2012, in press; Daisley et al., 2010; Mascalzoni et al., 2010; Regolin et al., 2011; Vallortigara, 2012). With regard to fish species, a related approach can be found in the work of Rui Oliveira. This research is centered on the study of social competence and of the cognitive processes involved in it, with an integrative approach and a particular focus on the zebrafish as an animal model (Oliveira, 2012; Taborsky and Oliveira, 2012). Among other things, these studies aim to understand how the brain translates social information into flexible behavioral responses, how this impacts on individual fitness, and how this process is constrained by the individual developmental history or by trade-offs with other adaptive competences (Taborsky and Oliveira, 2012). Teleost fish represent an ideal model to identify basic information processing mechanisms that provide the functional building blocks of social behavior across different species with varying social systems. In fact, among teleosts we have a pronounced diversity of social systems in closely related species. This allows for planned phylogenetic comparisons of perceptual and cognitive abilities. Moreover, model species such a zebrafish also offer genetic tools for the study of selected neural circuits (Oliveira, 2012), making this a most promising field of research for future interdisciplinary studies.

Future studies should thus capitalize on the potential insights offered by fish species to understand the evolution of the vertebrate visual system, especially by further investigating the neural correlates of perceptual organization in species belonging to distant taxa. On this regard, an important aim for future work should be to increase our knowledge of the perceptual abilities of species specifically selected because of their informative value, based on their phylogenetic relation with other species of known perceptual abilities. A particular case is that of jawless fish (Agnatha), such as lampreys and hagfish, whose susceptibility to some fundamental perceptual phenomena has never been tested, despite their great phylogenetic interest.

## Conflict of interest statement

The authors declare that the research was conducted in the absence of any commercial or financial relationships that could be construed as a potential conflict of interest.

## Footnotes

^1^Interestingly, perception of subjective contours and neurons performing interpolation operations that can support them have been found also in insects (van Hateren et al., 1990; Horridge et al., 1992).

^2^In fact, variations in spectral composition of distant regions of the visual field affect the perceived color of an object as much as in nearby regions (Land, 1983; Land et al., 1983).
